# Total Knee Replacement: Subject-Specific Modeling, Finite Element Analysis, and Evaluation of Dynamic Activities

**DOI:** 10.3389/fbioe.2021.648356

**Published:** 2021-04-16

**Authors:** Iliana Loi, Dimitar Stanev, Konstantinos Moustakas

**Affiliations:** ^1^Department of Electrical and Computer Engineering, University of Patras, Patras, Greece; ^2^School of Engineering, Institute of Bioengineering, École Polytechnique Fédérale de Lausanne, Lausanne, Switzerland

**Keywords:** total knee replacement, finite element, subject-specific modeling, musculoskeletal, sensitivity analysis

## Abstract

This study presents a semi-automatic framework to create subject-specific total knee replacement finite element models, which can be used to analyze locomotion patterns and evaluate knee dynamics. In recent years, much scientific attention was attracted to pre-clinical optimization of customized total knee replacement operations through computational modeling to minimize post-operational adverse effects. However, the time-consuming and laborious process of developing a subject-specific finite element model poses an obstacle to the latter. One of this work's main goals is to automate the finite element model development process, which speeds up the proposed framework and makes it viable for practical applications. This pipeline's reliability was ratified by developing and validating a subject-specific total knee replacement model based on the 6th SimTK Grand Challenge data set. The model was validated by analyzing contact pressures on the tibial insert in relation to the patient's gait and analysis of tibial contact forces, which were found to be in accordance with the ones provided by the Grand Challenge data set. Subsequently, a sensitivity analysis was carried out to assess the influence of modeling choices on tibial insert's contact pressures and determine possible uncertainties on the models produced by the framework. Parameters, such as the position of ligament origin points, ligament stiffness, reference strain, and implant-bone alignment were used for the sensitivity study. Notably, it was found that changes in the alignment of the femoral component in reference to the knee bones significantly affect the load distribution at the tibiofemoral joint, with an increase of 206.48% to be observed at contact pressures during 5° internal rotation. Overall, the models produced by this pipeline can be further used to optimize and personalize surgery by evaluating the best surgical parameters in a simulated manner before the actual surgery.

## 1. Introduction

Osteoarthritis is a degenerative disease that affects millions of people every year and has unprecedented consequences on their quality of life. Total knee replacement (TKR) or total knee arthroplasty (TKA) is mainly used as a surgical treatment of osteoarthritis since it relieves pain, improves mobility (Woolhead et al., 2005; van Kasteren et al., 2018), and is a common practice usually among female patients at the age of 65 (Woolhead et al., 2005; Kim et al., 2014; Lum et al., 2018; van Kasteren et al., 2018). However, pain and immobility issues insist on a 10–34% of TKR patients (van Kasteren et al., 2018) caused by implant failure. Among the most prevalent causes of implant failures are loosening, instability, malalignment, polyethylene wear of tibial insert, osteolysis, and infection (Mulcahy and Chew, 2014; Lum et al., 2018; Postler et al., 2018), which would lead to revision surgery. Remarkably, the life span of a TKR implant is around 10 years on average (10–15 years implant survival rate is more significant than 90% as reported in Pang et al., 2016; Su et al., 2020 and survival rates of 95% at 15 years as mentioned in Moewis et al., 2018) and sometimes reaches 15–20 years, after which a TKR revision operation is almost necessary.

Most studies concerning TKR use finite element analysis (FEA) to further analyze contact pressures in TKR implants and investigate the causes of implant failures. Especially, studies focus on stresses at the joint, i.e., between the femoral component and tibial insert to reveal the effects of implant materials, sagittal radius, flexion angle (Shashishekar and Ramesh, 2007), various implant designs (Usman and Huang, 2018), surgical decisions (Su et al., 2020), or misalignment (Pang et al., 2016; Moewis et al., 2018) on the function of the knee joint. Other scientific articles on FEA focus on developing custom-made knee implants (Sun et al., 2015; Li et al., 2017) or conducting “what if” studies about implant positioning during TKA (Mou et al., 2018). Moreover, studies, such as Stanev et al. (2016, 2020) and Benos et al. (2020) that use musculoskeletal and finite element (FE) models of the knee to predict surgery's effect in a pre-operative manner might be an interesting extension of this work for TKR. The methodologies presented in Benos et al. (2020) and Stanev et al. (2020) focus on finite element simulations for evaluating anterior cruciate ligament (ACL) reconstruction surgeries, thus, they are engaged with FEM for non-implanted knees. These procedures were enriched and used to develop FEMs representing the knee joint after a TKA operation.

The role of sensitivity analysis in FE modeling to study the load distributions either at the tibiofemoral joint or at the implant-bone contact areas is also addressed in TKR literature, with implant malalignment attracting the most scientific attention. Many studies try altering TKR implants' alignment with respect to the bones to study the sensitivity of contact pressures, Von Mises stresses, strains, and ligament balance. In particular, the measurement of contact pressures in the tibial plateau (tibial insert) of a retrieved custom made knee prosthesis in Sun et al. (2015) showed that the developed contact pressures are sensitive to high flexion angles (e.g., 135°) during squatting on kneeling that can lead to material loss at the posterior region of the tibial insert. Accordingly, in Liau et al. (1999) three commercial knee prostheses were used for testing under a compression load at flexion of 0 and 10°, in both standard and malalignment conditions in order to study the effect on contact pressures. The same authors also investigated the effect of malalignment on contact stresses in the tibial component by evaluating 3D TKR finite element models (FEMs) for three different implant designs under three malalignment conditions (medial translation, internal rotation, and varus tilt of the femoral component relative to the tibial component) (Liau et al., 2002). In the work of Innocenti et al. (2016), three TKA models with different varus/valgus alignment were created and compared with a neutrally aligned one to study the effect of deviations from neutral alignment on bone and implant stresses and on ligament strain. In order to do so, the authors changed the alignment of either the femoral component or tibial insert or both implant parts simultaneously. The effects of the femoral component's misalignment in the sagittal plane on the Von Mises stress and contact pressure distribution of the tibial component were investigated in Pang et al. (2016) by using FEA on a retrieved custom-made knee prosthesis after the fracture of the tibial tray component. Moreover, in Yang et al. (2020) different TKR FE models were developed with changes in tibial tray-bone alignment, tibia marker locations, and friction coefficient to investigate possible sensitivities in the tibial tray–bone micromotions (Yang et al., 2020). Another example is (Moewis et al., 2018) where the sensitivity of stress and strain distributions on two FE models, one with a physiological prosthesis and one with a horizontal implant, to normal positioning of the components and internal and external mal-rotation of the tibial component was investigated. In this study, we also perform a sensitivity analysis to quantify the effect of ligament and positioning parameters on the tibia's contact stresses.

Frameworks for modeling computational models that also provide methods for evaluating the proposed workflows' validity are outlined in studies (Kiapour et al., 2014; Marra et al., 2015; Navacchia et al., 2016; Shu et al., 2018). The study of Navacchia et al. (2016) presents a methodology to estimate the joint contact mechanics in patients after TKA, during a plethora of daily life activities, such as gait, walking downstairs, and chair sit through TKR FE models. Kiapour et al. (2014) aimed at developing and validating a FEM of the lower extremity with an anatomically accurate representation of the knee joint to evaluate tibiofemoral biomechanics and injury mechanisms, especially investigating ACL injury. Furthermore, Shu et al. (2018) developed a pipeline for producing a subject-specific FE-musculoskeletal model of human right lower implanted extremity that combines the interactions between the prosthetic mechanics and multibody dynamics after a TKA surgery in order to predict muscle-tendon forces, joint contact forces, and contact mechanics of prostheses. Similarly, Marra et al. (2015) presented a musculoskeletal modeling framework to develop subject-specific models, which can simultaneously estimate *in vivo* ligament and muscle forces, tibiofemoral contact forces, and knee joint kinematics. A similar approach was used in our framework as well, although special attention is given to the automation of the FEM developing process, boosting performance, and enhancing application potential.

Many of these studies are developing their FEMs manually by using open-source software or by obtaining commercially available generic models. Furthermore, TKR FE models' validation is a quite critical and complex procedure with only a few studies in current literature dealing with it, such as Kiapour et al. (2014), Marra et al. (2015), Woiczinski et al. (2016), and Shu et al. (2018). Particularly, in Woiczinski et al. (2016) knee cadaveric experiments were realized for the validation of the FE model that emulates the knee joint and sensitivity analyses of Young's modulus of cartilages and ligaments. However, motion capture data combined with FEA simulations are preferred over unobtainable knee rigs for validating FEMs. The time-consuming and complicated procedure to develop and validate the patient-specific FEM poses an obstacle to conducting kinematic, dynamic, and sensitivity analyses on post-operational implanted knees. Thereafter, the need for a framework that would produce subject-specific FEMs emerges.

This study aims to establish a framework for building and validating subject-specific FE knee models that can be used to analyze the knee's mechanical behavior. The importance of patient-specific musculoskeletal FEMs was highlighted in Xu et al. (2020), showing that individual bone characteristics, such as size and shape, affect joint kinetics and load distribution estimations at the tibial bone. In the proposed framework, the FEM is constructed in a semi-automatic manner, and the users can easily personalize their FEMs by altering the implant-bone geometries and the alignment between them, as well as other parameters of the model (e.g., rigid constraints, implant material, and contact interface properties).

Model validation is the Achilles' heel of the FE models. In this study, we make use of the 6th SimTK Grand Challenge Competition (SimTK GCC 2014) (Fregly et al., 2012) in order to validate the contact pressures and forces for different movements. Furthermore, the validation procedure includes the observation and measurement of contact pressures on the surface of the tibial insert in relation to gait patterns and the estimation of contact forces at the tibiofemoral joint. Similarly to Marra et al. (2015), Woiczinski et al. (2016), and Shu et al. (2018), the articular forces were compared to *in vivo* measurements recorded by the implant sensor, called e-Tibia (Fregly et al., 2012). In this way, the use of hard to find knee rigs can be decreased.

The proposed framework can be used to define subject-specific TKR FE models, conduct “what if” studies and sensitivity analyses. The FEM produced by this pipeline can be used to organize a personalized surgery by evaluating the best surgical parameters via simulation before the TKA. A surgeon may experiment with various implant-bone alignments and possible bone resections while developing their individualized model through the proposed framework, always aiming for the optimal/least load distributions at the tibiofemoral joint. Implant-bone positioning significantly affects contact pressures at the joint since a minimal discrepancy within 2–3° between various alignment procedures might result in a poor post-operative outcome, increased implant stress, and decreased implant survivorship as indicated in Lording et al. (2016), Schiraldi et al. (2016), and Riviére et al. (2018).

Finally, many notable studies approach the aspects of modeling and simulation in TKR (Kiapour et al., 2014; Marra et al., 2015; Navacchia et al., 2016; Shu et al., 2018). While they outline the blueprints for modeling and validation, the findings are difficult and hard to reproduce. One of this paper's goals is to outline the modeling choices and automate this procedure using open-source tools, such as FEBio (Maas et al., 2012), OpenSim (Seth et al., 2011, 2018), and Python packages. The benefits are that if a new subject is to be studied, one can use the same scripts to create the FE model and perform simulations. This can aid in the reproducibility and sharing of models and simulations. To summarize, the contributions of the proposed framework are as follows:

- Automatically build FEMs that can be used for evaluating knee mechanics after TKR.
- Validation of a personalized FEM using different subject-specific gait trials provided in 6th GCC data set.
- Perform sensitivity analyses to assess the influence of modifications in ligament properties and implant positioning on tibiofemoral contact pressures and investigate possible uncertainties on the produced models.

## 2. Methods

**Figure 1:**
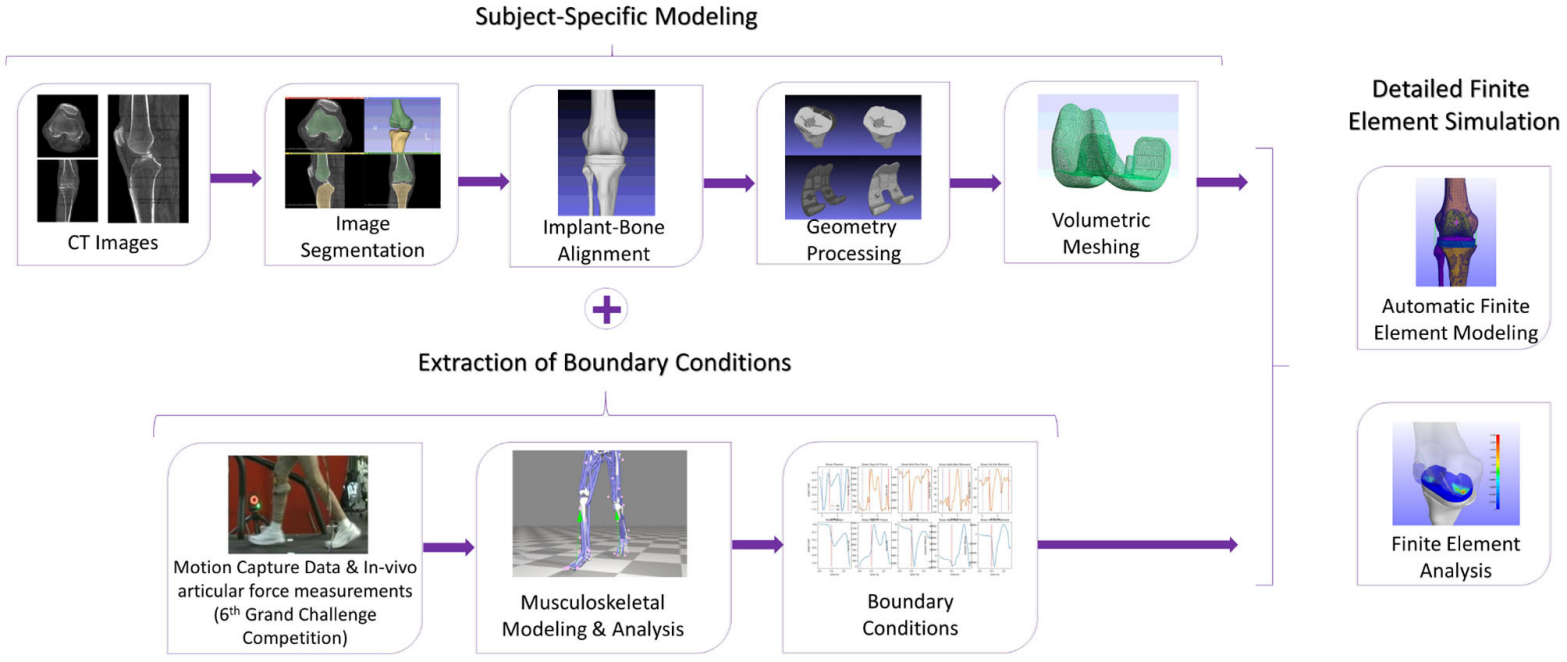
The methodology to develop a subject-specific total knee replacement finite element (TKR FE) model. The subject-specific modeling includes the segmentation and aligning processes as well as geometry processing and volumetric meshing. The TKR model will have boundary conditions extracted from the 6th GCC, and it will be used for finite element analysis (FEA).

In Figure 1, the workflow of the proposed semi-automatic pipeline is depicted. The FE construction procedure includes reconstructing the knee bone surfaces through image segmentation techniques on patient-specific pre-operational Computed Tomography (CT) data (Benos et al., 2020; Stanev et al., 2020). Many studies, like Woiczinski et al. (2016), Li et al. (2017), Moewis et al. (2018), Nikolopoulos et al. (2020), Su et al. (2020), and Xu et al. (2020) that aim at developing patient-specific FE models, adopt this method. These 3D bone models were later aligned with the knee joint's implant components by using segmentation software on post-operative CT images. The segmentation and alignment procedure through image processing software requires 20–30 min by following the guidelines presented in section 2.2 (~10–15 min for segmentation and about the same time for alignment). Furthermore, 3D processing software [Meshlab (Cignoni et al., 2008), Meshmixer (Schmidt and Singh, 2010)] was used to correct geometry artifacts that cause convergence problems. Isotropic [Instant Meshes (Jakob et al., 2015)] and volumetric [TetGen (Si, 2015)] re-meshing of the implant-bone geometries must be carried out so that the correct computation of load distributions at contact tractions is ensured. Geometry preparation, including geometry preprocessing, isotropic and volumetric re-meshing of all bone, and implant geometries, requires around 5 min and is not automated.

Prediction of the patient's gait characteristics after the operation can be performed through post-operative gait data sets (i.e., video motion data) provided by the 6th GCC. The resulted gait characteristics are used in the musculoskeletal modeling tool OpenSim. The extracted boundary conditions from the patient's different gait patterns contributed to the validation of the FEM since the 6th GCC contains the ground truth contact forces measured by the implanted sensor. The extraction of boundary conditions procedure is automatic. It can be completed in about 1-min max by using OpenSim tools and simple parametric scripts to preprocess the Grand Challenge data (conversion from C3D to OpenSim formats, reorientation of data's marker positions, rotation of ground reaction force vectors, data filtering, and conversion of marker data units in order to match OpenSim's coordinate and measurement system units).

The development of the FEM of the implanted knee is fully automatic and is realized through Python scripts in <3 min or even <1 min for simplified meshes. Finite element analysis of the model was carried out through FEBio software (Maas et al., 2012). The analysis needs around 2–2 and a half hour to complete in a Windows 10 64-bit operating system with Intel Core i7 3.5 GHz processor and an 8 GB RAM. Regarding the above procedures, the overall pipeline needs around 3 h from anatomical segmentation to output analysis. However, if we were to model our FEM manually, we would need at least 2–3 h, almost doubling the overall time (5–6 h).

This framework was used to study the sensitivity of contact pressures at the tibiofemoral area by performing changes in ligament properties and implant positioning. The parameters used for the sensitivity study were the LCL and MCL origin point positions, stiffness and reference strain, as well as the alignment of the femoral component and tibial insert with respect to knee joint bones. By altering these parameters, we can study how contact pressures are affected. Thus, we provide valuable information to clinicians for pre-surgical optimization and personalized surgery design. Overall, a sensitivity analysis was used to quantify the uncertainty in the model and identify parameters that can influence the results of FEMs.

### 2.1. Description of the Data Set

The 6th GCC data set is intended to aid in creating and validating computational knee models to predict articular contact, muscle, and ligament forces during gait or any other movements (Fregly et al., 2012). These data come from patients with knee implants that provide direct contact force measurements in the knee (e-Tibia). Specifically, it contains measurements obtained from a male patient's right implanted knee, with a height of 172 cm and a weight of 70 kg. The patient appears to have a valgus leg alignment. The 6th Grand Challenge data set is publicly available, and institutional review board approval and subject informed consent were obtained for all de-identified competition data being released by the research team of Fregly et al. (2012).

Motion capture data, including marker positions, GRF, and EMG, were provided for various movements. These raw data are recordings of the subject's normal gait (“ngait_og”) pattern plus bouncy (“bouncy” —overground gait trials with clean force plate strikes using a bouncy gait pattern), crouch (“crouch_og” —overground gait trials with clean force plate strikes using a crouch gait pattern), forefoot strike (“mtpgait” —overground gait trials with clean force plate strikes using a forefoot strike gait pattern), and smooth (“smooth”) gait patterns. The data were used for musculoskeletal modeling to extract boundary conditions for the FEM, and they have the same unit measurements: mm for marker position data, N for forces, mm for the center of pressure (COP), and N-mm for moments. Moreover, tibial contact force recordings from the implant's sensor (e-Tibia) are provided for a subset of gait trials and used to evaluate contact force estimates calculated by FEA. The e-Tibia measurements include forces, moments, goniometer synchronization signal, and vertical ground reaction force synchronization signal sampled at a frequency of 120 Hz. The experimental set-up's reference directions for overground trials were the z-axis being the vertical direction, x-axis facing forward, and y-axis facing the patient's right direction.

Finally, patient-specific pre-operative and post-operative CT scan images were provided for the knee region (pre-op) and entire leg (post-op—hip to ankle), as well as patient-specific implant-bone geometric models of the subject's femur patella, tibia, and fibula. Pre-operative CT images were used to extract subject-specific bone geometries and the patient's post-operative CT data to align the joint bones with the knee implant parts. Thus, only the implant components for the subject's implanted leg were utilized in the FE model, namely femoral component, a tibial insert, and tibial tray.

### 2.2. Segmentation and Alignment of Implant-Bone Geometries

**Figure 2:**
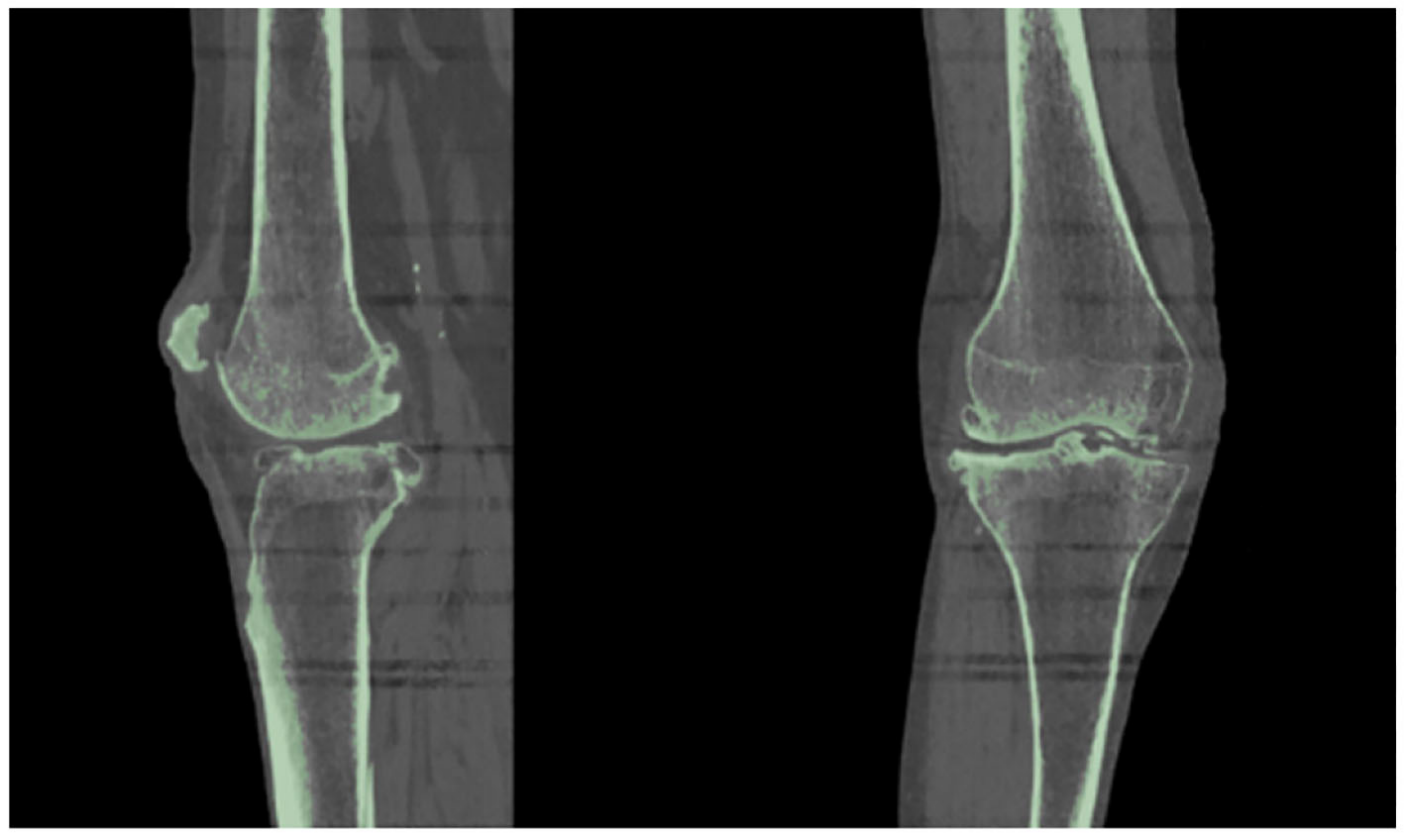
Results obtained using 3D Slicer's “Threshold” algorithm. Since the bones have the same intensity in the CT image, the software corresponds one label (indicated with green color) to all the pixels representing the patient's knee bones.

**Figure 3:**
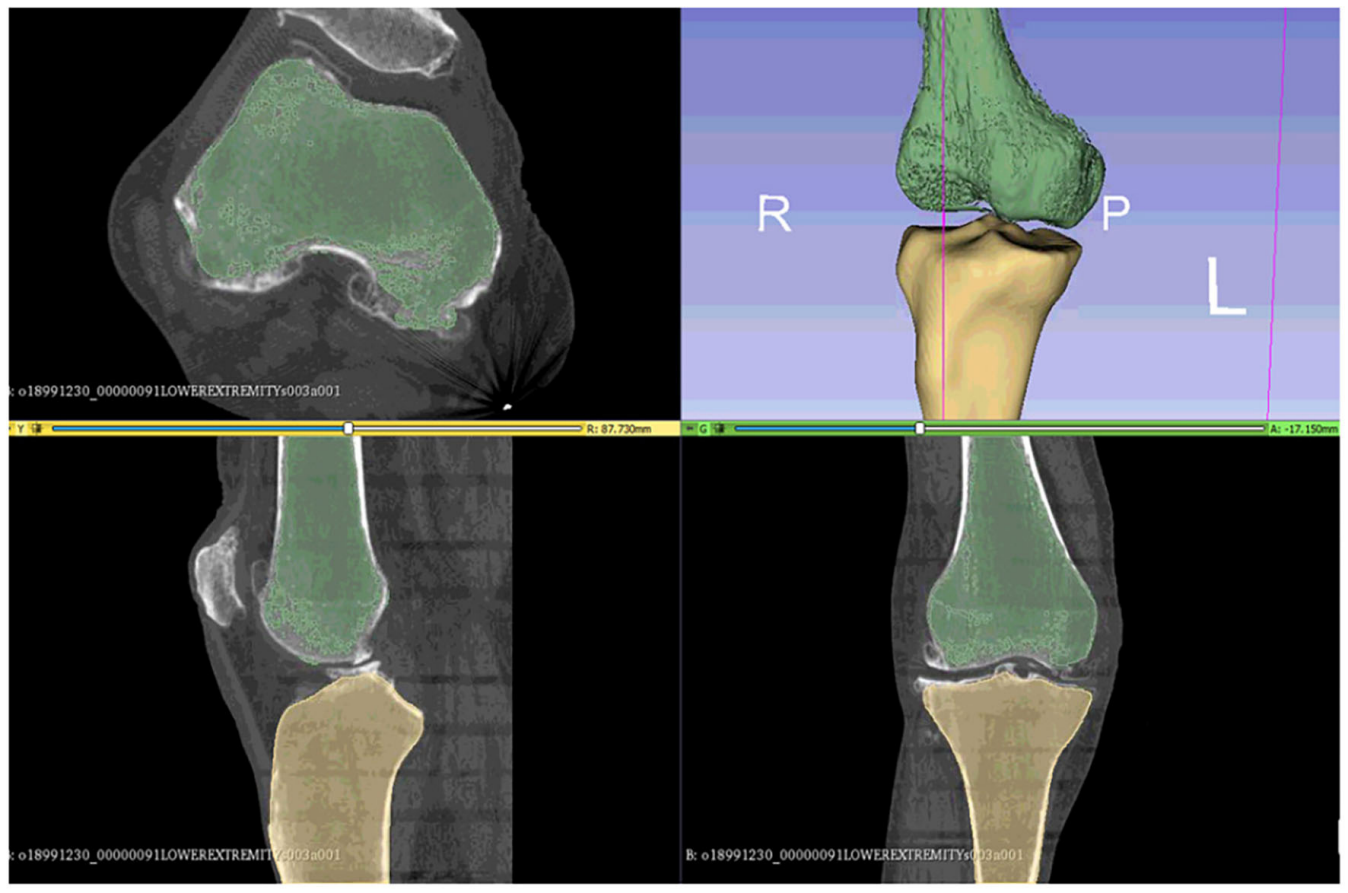
The femur can be differentiated from the tibia and the tibia from fibula using the 3D Slicer's algorithms that can separate regions corresponding to the same label.

As suggested in a lot of studies (Kiapour et al., 2014; Marra et al., 2015; Woiczinski et al., 2016; Li et al., 2017; Moewis et al., 2018; Benos et al., 2020; Nikolopoulos et al., 2020; Stanev et al., 2020; Su et al., 2020; Xu et al., 2020), the best way to obtain a patient-specific 3D representation of the knee joint bones is to segment them from MRI or CT image data obtained from the subject under examination. For doing so, a segmentation method was performed with 3D Slicer (Kikinis et al., 2014) to extract the geometries of the knee bones before TKA, using subject-specific pre-operational CT scans obtained from the 6th GCC data set. The segmentation algorithm provided by 3D Slicer corresponds labels to voxels representing specific biological structures, i.e., knee joint bones, of the region depicted in the 3D CT image, depending on a chosen threshold value. An example of corresponding labels to bone regions is illustrated in Figure 2. Since the voxels representing bones in the CT images are of the same intensity, the algorithm corresponds the same label to all bony areas, and, thus, no separation between bones is apparent. This problem can be easily solved by an algorithm that creates a unique label value for each connected region in the initial label map, also provided by 3D Slicer. The result after separation, where the femur is distinguished from the tibia, is depicted in Figure 3.

Then, the model's implant-bone alignment must change to simulate one of the understudied subjects. In every patient, after TKA, knee bone alignment may vary according to the implant positioning or to medical interventions that would be performed to correct possible malalignment due to osteoarthritis. Thus, implant component positioning greatly affects the load distribution in both realistic and FEM knees (Liau et al., 1999, 2002; Kiapour et al., 2014; Marra et al., 2015; Sun et al., 2015; Lording et al., 2016; Pang et al., 2016; Schiraldi et al., 2016; Moewis et al., 2018; Riviére et al., 2018; Xu et al., 2020; Yang et al., 2020). To improve the alignment, it is necessary to position the segmented 3D bone geometries with the implant parts being available in the GCC data set using the patient's post-operative CT images. Through the CT scans, we can accurately determine the alignment landmarks and anatomical axes of the lower limb as supported in Victor et al. (2009). The alignment process was carried out in the 3D Slicer tool.

**Figure 4:**
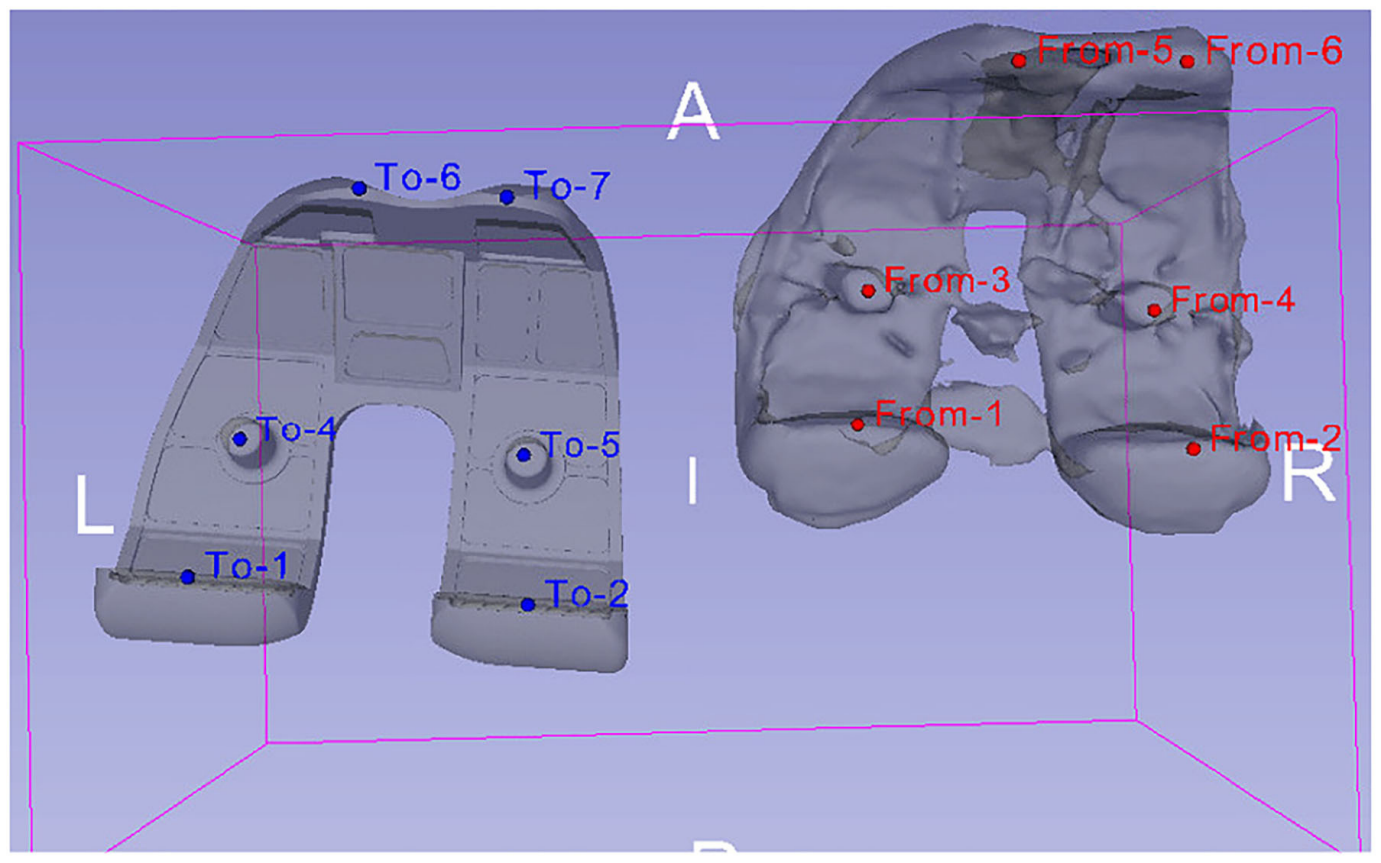
Alignment of femoral component's 3D model (left—obtained from 6th GCC data set) with a trial model (right), which was produced through the segmentation of patient's post-operative CT images. The alignment was carried out by comparing the position of landmarks placed on the trial model (red “From” points) to the ones placed on the femoral component's 3D geometry (blue “To” points).

The registration algorithm that 3D Slicer provides fits data acquired from an object (e.g., a trial model of femur bone segmented from post-operative image data) to another data set (e.g., femur bone geometry), which is gathered from the same object under different conditions (Kikinis et al., 2014). Specifically, trial models of the bones were created from post-operative CT scans, and their position was compared with the position of the segmented bone geometries. This comparison was made using landmarks (“fiducial points”), which were placed manually in key positions on CT images of the femur and tibia. In Victor et al. (2009), it was shown that variations in the registration of bony landmarks and anatomical axes on the CT images of the lower limb due to observer's expertise do not greatly affect the accuracy of locating reference points and corresponding axes. Thus, it is safe to assume that locating landmarks' precision is slightly affected by manually determining alignment landmarks on CT images of the femur and tibia. The above procedure results in the 3D bone meshes being translated from their original position to CT image coordinate system, where the trial models reside. An example of aligning the femoral component's 3D geometry using a trial model is illustrated in Figure 4.

### 2.3. Geometry Preparation

Geometry processing methods are utilized to correct artifacts on both the implant and bone 3D geometries of the FEM, which would induce model convergence problems. More specifically, the 3D knee joint bone geometries were segmented from the patient's CT data collected before the TKR operation. These geometries do not have the incisions made from the implant placement. Thus, they need to be cut in order to “fit” the implant parts appropriately. Boolean functions were used for cutting the femur and tibia bone geometries. Subsequently, bone and TKR implant geometries were tested individually for solving 3D model singularities. Features like holes, self-intersections, duplicated vertices, small components, and many more were then filled or removed either manually or automatically by using algorithms provided by open-source software, such as Meshlab (Cignoni et al., 2008) and Meshmixer (Schmidt and Singh, 2010).

**Figure 5:**
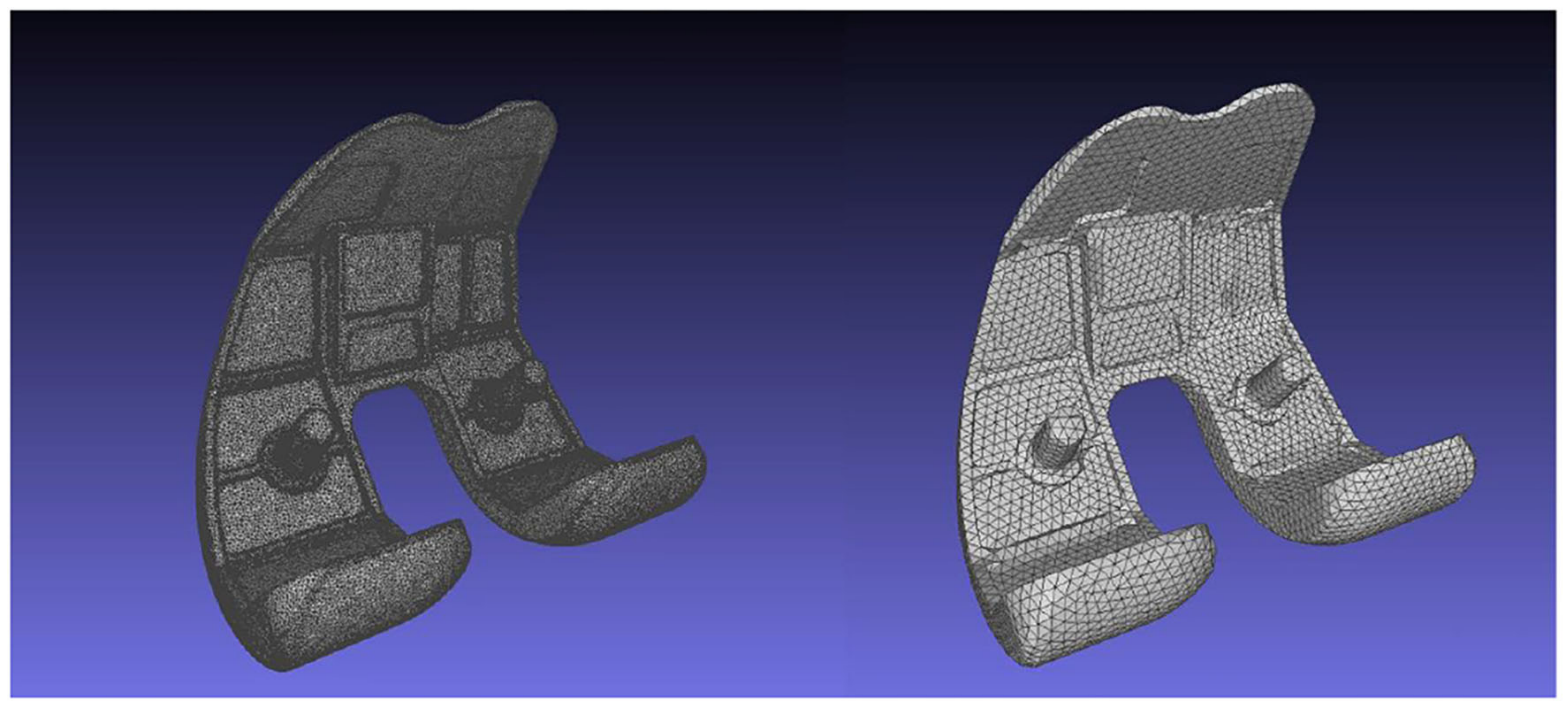
Femoral component's mesh before (left, 99.995 vertices), and after (right, 6.000 vertices) the Instant Meshes algorithm.

The artifact correction process on the 3D models would cause the creation of an irregular mesh, something which is undesirable in FEA. As a result, the 3D geometries consisting of the TKR model were isotropically re-meshed. The Instant Meshes algorithm (Jakob et al., 2015) was utilized for the isotropic re-meshing and simplification of the instrumented knee geometries. The algorithm results in creating re-meshed geometries with elements of the same size and area. The element count of the FEM's geometries can also be handled through Instant Meshes. The reduction of the TKR geometries' complexity will minimize both the computational cost of the finite element calculations and the procedure's overall running time. For example, the femoral component 3D model included in the 6th GCC data set has a total of 99.995 vertices, a number that can be minimized down to 6.000 through isotropic re-meshing, without affecting geometry's shape detail (Figure 5). The geometries of all FEM parts were simplified to have the same mesh density.

Finally, both the geometries created through segmentation and the ones acquired from the 6th GCC are STL files, meaning that they consist of a 3D triangulated mesh with a hollow interior. Thus, their volumetric meshes must be created since, in FEA, internal stresses and forces throughout the entire volume of a mesh are computed. The creation of the geometries' volumetric meshes was realized through Delaunay-based algorithms implemented by Tetgen (Si, 2015), which produces a constrained tetrahedral mesh. Similar to the Instant Meshes algorithm, Tetgen could not create the volumetric mesh of geometry with singularities. The geometry preprocessing methods used in this project succeed in creating meshes that can be processed by the above Delaunay-based algorithms.

### 2.4. Implant Parts and Material Properties

Python scripts were developed for the automation of the FEM development procedure. FEMs were created according to the FEBio FEM format. These scripts essentially produce an XML (FEB) file including all the characteristics that a FEM representing an instrumented knee joint must-have. Constitutive and loading parameters, such as the pre-processed subject-specific geometry data, materials, contact interfaces, boundary conditions extracted from OpenSim analyses, and joint connectors, were defined in the XML file.

**Figure 6:**
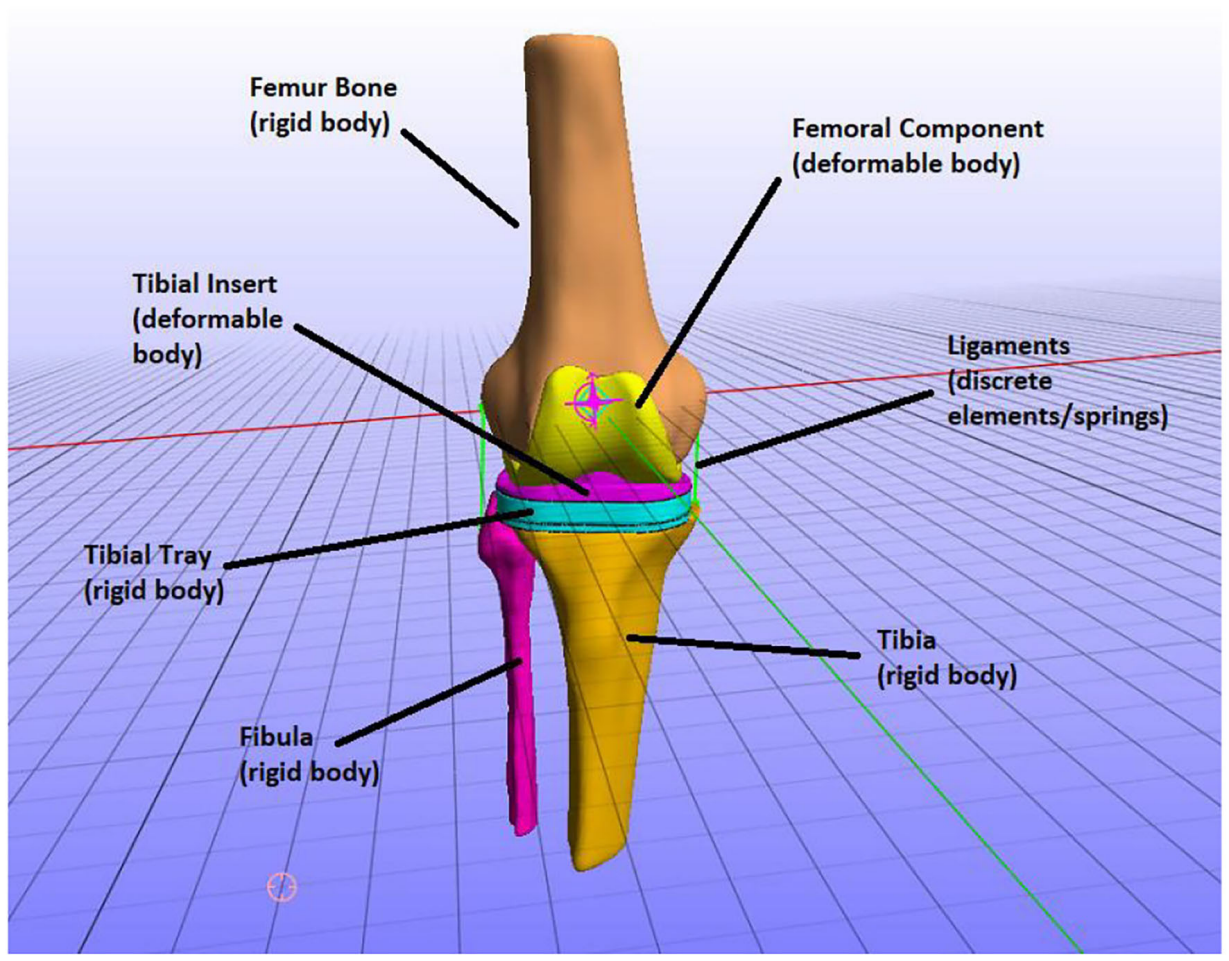
The parts of the finite element (FE) model, i.e., knee bones and implant parts and how they were modeled (e.g., the tibia was modeled as a rigid body).

The FEM consists of six parts: the three bones that constitute the knee joint—femur, tibia, and fibula—and the three parts of the knee implant—femoral component, tibial insert, and tibial tray. The knee implant originating from the 6th GCC was based on a Zimmer Natural Knee II design. Each one of these parts is illustrated in Figure 6.

In the TKR model, the knee joint bones were modeled as rigid bodies. In natural knee joint (without an implant) studies, it is typically assumed that bones are rigid to study the mechanics of soft tissues (Madeti et al., 2015; Benos et al., 2020). That is because bones have a much higher Young's modulus value than the modulus of elasticity of the soft tissues, and thus the deformation of bones is minimum to zero compared to one of the cartilages. However, if stresses on bones were to be calculated, the bones can be simulated as linear elastic and are usually divided into cortical and cancellous parts for more realistic representation (Benos et al., 2020). In this study, we are not interested in calculating the stresses on the bones but the implant mechanics (e.g., contact pressures on tibial insert). Therefore, the bony geometries of the model are considered rigid bodies.

We explored the option of modeling the bones as deformable bodies. The material properties used were Young's modulus = 13.4 GPa and Poisson's ratio = 0.24 (Benos et al., 2020). The model was provided boundary conditions from normal gait (ngait_og1). A tied elastic contact interface was defined between the femur and femoral components. The deformable model produces the same stresses on the tibial insert, although differences were observed in 1st and 2nd peak contact pressures (Table 1). The contact pressure of the 1st peak is 2.93 MPa for the rigid bone model and 2.98 MPa for the deformable bone model. The contact pressures of the 2nd peak are 6.22 and 6.52 MPa, respectively. As for the COP, no significant differences were observed. Modeling the bones as deformable seems more natural; however, it can significantly increase the execution time (5 h compared to 2 h for the rigid bone model). This can influence the number of simulations performed for the sensitivity analysis. We decided to perform the sensitivity analyses using the simplified rigid bone model because it requires less time to execute a simulation.

**Table 1:** Maximum contact pressures during patient's different gait patterns.

**Gait trial**	**1st Axial peak force (Mpa)**	**2nd Axial peak force (Mpa)**
Ngait_og1	2.93	6.22
Mtpgait2	3.21	7.74
Crouch_og2	4.39	5.06
Bouncy4	3.64	7.85
Smooth1	2.19	3.80

The tibial tray was also modeled as a rigid body since it has a much higher Young modulus than the tibial insert. In the cited literature, both the tibial tray and tibial insert are modeled as deformable bodies. It is usually considered for the tibial tray to have metallic materials, whereas polyethylene is often used for modeling tibial insert. Therefore, the tibial tray presents minimum deformation during knee function (Pang et al., 2016; Usman and Huang, 2018) compared to the tibial insert on which most loads are applied. Consequently, since femur, tibia, fibula, and tibial tray were modeled as rigid bodies, they can be defined using the rigid material model where nodal degrees of freedom are reduced down to six (Maas et al., 2012).

The rest of the TKR implant parts, namely the femoral component and tibial insert, were modeled as deformable bodies (Figure 6). That means that they are composed of elastic materials and are assigned to compressible neo-Hookean materials, which have a non-linear stress–strain behavior. More specifically, a compressible Neo-Hookean constitutive model for a solid material has a strain-energy density, which is derived from:

(1) $$\begin{array}{l} \text{Ψ}=\frac{G}{2}\left(I_{1}-3\right)-G\ln J+\frac{\lambda }{2}\ln J^{2}, \end{array}$$

where the constants *G* and λ are material coefficients, *I*_1_ = *tr***C** is the first invariant of the right Cauchy–Green deformation tensor **C**, and *J* is the determinant of the deformation gradient tensor. Equation (1) is solved by FEBio, and the material properties given will be Young's modulus *E* and Poisson's ratio *v*, where *E* = 2*G*(1+*v*) and λ = 2*Gv*/(1−2*v*) (Zimmerman and Ateshian, 2018). Thereafter, no other parameters need to be specified except linear parameters, such as Young's modulus and Poisson's ratio.

Consequently, it was considered for the metallic femoral component to have cobalt-chrome-molybdenum alloy (CoCrMo, ISO5832-4, Young's modulus: 210,000 MPa, Poisson's ratio: 0.3) and ultra-high molecular weight polyethylene for the tibial insert (UHMWPE, ISO5834-2, Young's modulus: 1,200 MPa, Poisson's ratio: 0.46), the same materials used in Moewis et al. (2018).

### 2.5. Ligament Modeling

During TKA, some ligaments are removed and replaced by the implant. The remaining ligaments after TKR are the collateral ones, namely MCL and LCL. The MCL and LCL ligaments are modeled as 1D tension-only non-linear spring elements, meaning that they cannot resist compression or shear but can sustain only tensile loads (Galbusera et al., 2014). These elements simulate non-linear force–strain behaviors until a threshold strain and have linear behavior afterward. The mathematical expression of this force–strain behavior was given by Blankevoort et al. (1991) and is modulated as follows:

(2) $$f=\left\{ \begin{array}{l} \frac{1}{4}k\frac{\epsilon^{2}}{\epsilon_{l}},\;0\leq \epsilon \leq \epsilon_{l}\\ k\left(\epsilon -\epsilon_{l}\right),\;\epsilon >2\epsilon_{l}\\ 0,\;\epsilon <0 \end{array}\right.$$

where *f* is the axial force carried by the ligament, *k* is the stiffness parameter, *ϵ* is the strain, and 2*ϵ*_*l*_ is the threshold strain, which indicates the change from the quadratic to the linear region (Blankevoort et al., 1991; Galbusera et al., 2014).

The stiffness parameters and reference strains were extracted from Blankevoort et al. (1991): for MCL, the stiffness was set to 8,250 N and *ϵ*_*r*_ = 0.04 and for LCL *k* =6,000 N and *ϵ*_*r*_ = −0.05. Notably, reference strain, *ϵ*_*r*_, is the initial strain of each line element when the joint is at the reference position (full extension) given by:

(3) $$\begin{array}{l} \epsilon_{r}=\frac{\left(L_{r}-L_{0}\right)}{L_{0}}. \end{array}$$

Hence,

(4) $$\begin{array}{l} L_{0}=\frac{L_{r}}{\left(\epsilon_{r}+1\right)} \end{array}$$

where *L*_0_ is the zero load length (when the ligament first becomes taut) given by equation (4). Also, *L*_*r*_ is the reference length, i.e., the length of each line ligament element of the model's reference position (Blankevoort et al., 1991). Parameters *k*, *ϵ*_*r*_, and ligaments' origins positions were modified in the context of the sensitivity analyses presented in section 3.2.

### 2.6. Model Constraints

Three contacts were defined for the produced FE model. The femur bone and femoral component contact and the one between the tibial tray and tibial insert were designated as rigid interfaces. Since the tibial tray, tibia, and fibula are rigid bodies and both tibia and fibula cannot perform any movement in this FEM, no contacts need to be defined between the tibia and tibial tray and between fibula and tibia. For defining rigid interfaces, the user must specify a rigid material and the faces of the deformable object's elements that are in contact with the rigid mesh. Moreover, a sliding-elastic contact was defined between the two deformable parts of the FE TKR model, namely the femoral component and tibial insert. Sliding contact interfaces at the femoral component-tibial insert interface permits sliding between the two geometries but prevents them from penetrating each other (Maas et al., 2012; Zimmerman and Ateshian, 2018). The user must specify the two contacting surfaces' faces, namely the femoral component and tibial insert.

The FEM defines a knee joint to control all 6 degrees of freedom (DOFs) of the knee. Rigid body constraints were applied on FEM's rigid body parts to enable or disable the DOFs of the knee joint, according to the knee movement that we were interested in analyzing. Thus, the FEM has three DOFs enabled. The femur, followed by the femoral component due to the rigid interface that connects them, can move across the z-axis (joint-distraction motion). According to FEBio's coordinate system, it can also rotate around the x-axis (flexion–extension) and y-axis (varus–valgus/abduction–adduction movement). These three DOFs are enforced by boundary conditions extracted from the GCC data set, while z-axis rotation and x- and y-axis displacement are locked. Moreover, the tibia, fibula, and tibial tray are fully constrained.

However, for constraining the DOFs of deformable parts of the model, boundary conditions combined with connectors were used. Rigid joint connectors connect two rigid bodies by producing non-linear constraints between them and allowing motion only along the joint's DOFs. The instrumented knee joint and its function are simulated by three rigid cylindrical joint connectors, each representing a different femur bone movement on the tibia, i.e., flexion–extension, joint–distraction, and varus–valgus rotation. These prescribed displacements and force constraints have a load curve associated with them, which was specified from data obtained by OpenSim's kinematic and dynamic analysis of patient-specific gait trials.

### 2.7. Boundary Conditions

The validation and description of the motion of the FEM were based on boundary conditions for different movements from motion capture data. These data constitute different subject-specific gait patterns (normal, bouncy, crouch, forefoot strike, and smooth), which were available at the 6th GCC data set. The OpenSim tool (Seth et al., 2018) was utilized for extracting the boundary conditions. The generic model used throughout OpenSim's procedure is the “Gait1992” model, a three-dimensional, 19-degree-of-freedom computer model of the human musculoskeletal system. This model was created in Yamaguchi and Zajac (1989), Delp et al. (1990), and Anderson and Pandy (1999).

**Figure 7:**
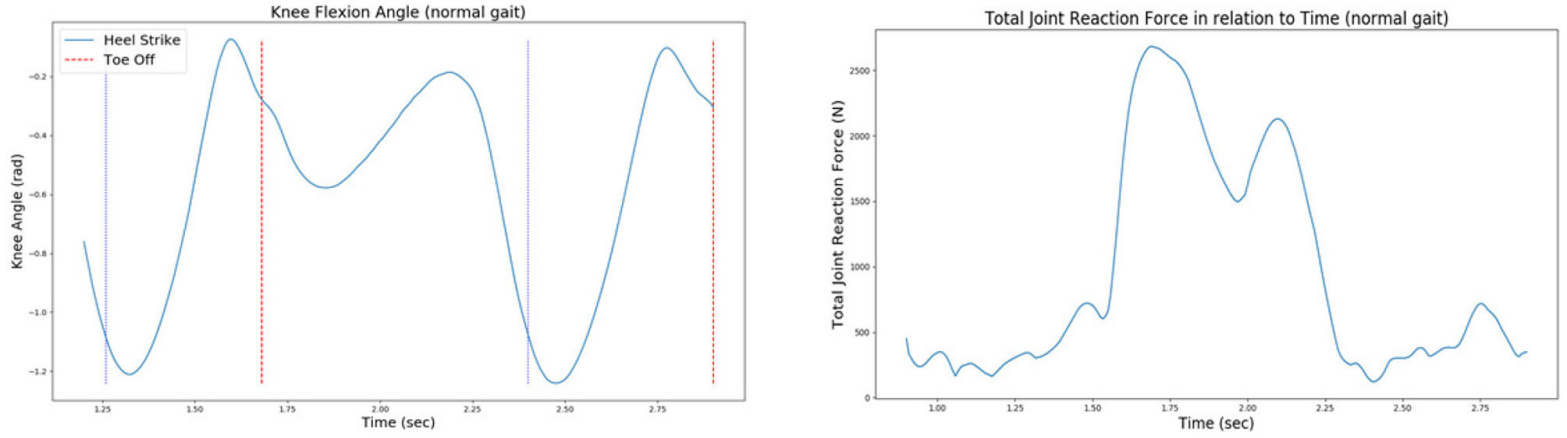
The knee joint angle **(left)** and the total joint reaction force **(right)** of the patient's right knee during normal gait, as extracted from the 6th GCC data set.

The OpenSim pipeline that must be followed consists of Scaling, Inverse Kinematics (IK), Static Optimization (SO), and Joint Reaction Analyses (JRA). Initially, the generic “Gait1992” model must be scaled to the subject's anthropometric measurements, and the model's (virtual) marker placement must be modified to coincide with the positioning of the markers used during the motion capture procedure. Subsequently, the IK tool computes in each time frame the error between experimental and virtual marker placement in order to position the model in a pose that best matches the experimental marker and coordinate values for that particular time step. The result obtained from IK is used by the SO tool to predict muscle forces at each time frame. These forces satisfy the experimentally measured movement, and they are then used to calculate reaction loads. The JRA tool computes resultant forces and moments at a joint. The desired measurements that need to be extracted and applied as boundary conditions to the models used for the validation of the proposed framework are as follows:

- The rotation angle of the knee joint during gait. This can be calculated through IK analysis.
- The forces and moments applied on the knee joint during gait. More specifically, the vertical (in the z-axis) knee joint reaction force drives the joint distraction/compression movement, while a y-axis knee moment drives the varus–valgus rotation. The values of forces and moments that cause the knee joint movement arise due to Joint Reaction Analysis (Figure 7).

It is worth noting that two simulation steps were defined for this project's FEM; an initialization step for ensuring the correct computation of contact tractions at the tibiofemoral joint and a second step where the actual simulation is realized. Explanatory, during the first step, femur bone starts moving vertically in the z-axis (longitudinal direction) until the first contact between the femoral component and the tibial insert is reached. Good contact between these two parts will yield correct load distribution estimations and minimize any convergence problems during FEA. Subsequently, at the second step, the femur is performing flexion–extension and adduction–abduction movements. The femur continues to move vertically during the second step so that femur-tibia contact is assured. The vertical movement is driven by a vertical knee joint reaction force extracted from GCC gait trials through OpenSim's analyses, as explained above. Moreover, flexion extension is driven by the joint angle extracted through IK, while a y-axis moment calculated by JRA drives abduction–adduction.

## 3. Results

A subject-specific FEM was created through the proposed pipeline. This model was validated in two steps. First, the tibiofemoral joint contact pressures were estimated through FEA and correlated with corresponding literature results. Also, contact forces at the femoral component-tibial insert interface were compared with *in vivo* sensor recordings of the 6th GCC data set.

The sensitivity analysis was conducted to understand the effect of alterations in both ligament parameters and implant component alignment on the distribution of contact pressures at the femoral component-tibial insert interface. More specifically, collateral ligaments (MCL and LCL) origin points, reference strain, and ligament stiffness, as well as implant-bone alignment, were used for the sensitivity study. Parametric scripts were developed to alter these parameters to create a plethora of FEMs for sensitivity analyses.

### 3.1. Comparison Between Predicted and Measured Contact Knee Loads

**Figure 8:**
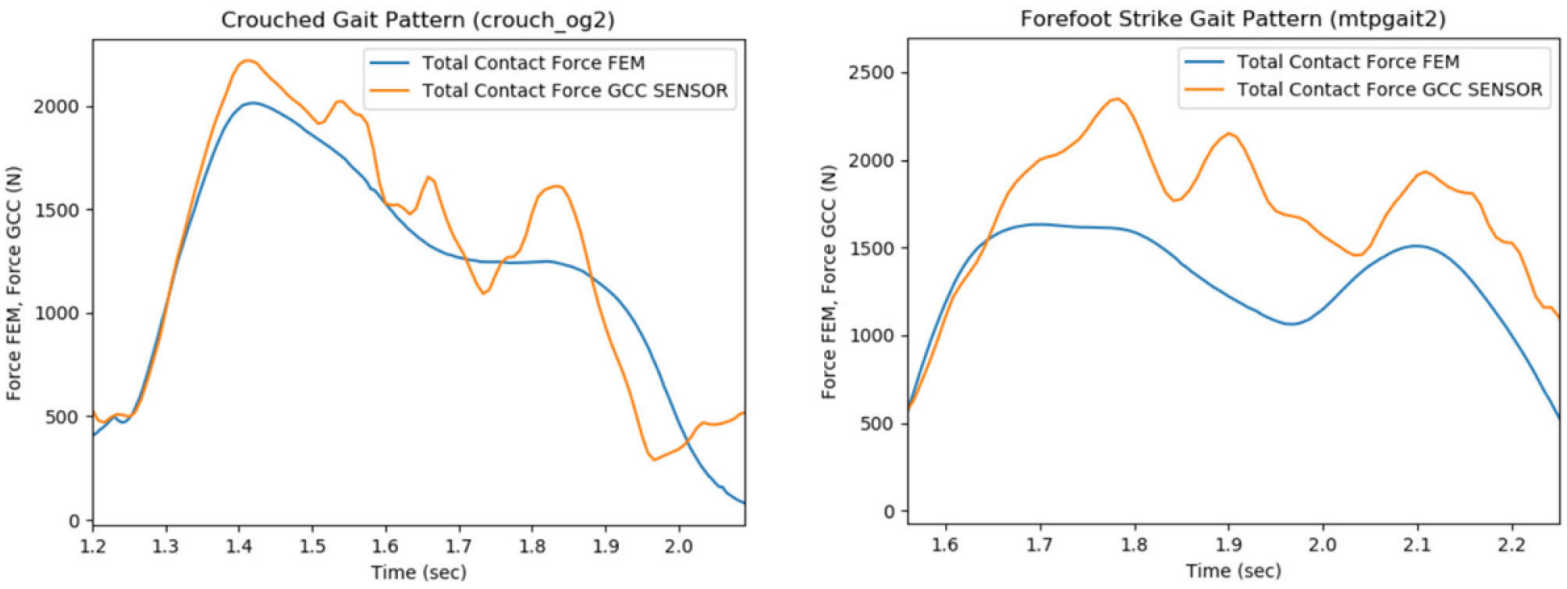
Comparison of the total contact force values that were recorded from the sensor of the implant (e-Tibia, GCC data set), which were provided by the 6th GCC (indicated with orange), with the estimated articular forces through the analysis of the total knee replacement (TKR) finite element model (blue curves), during the patient's crouch (crouch_og2, left plot) and forefoot strike (mtpgait2, right plot) gait patterns.

The FE model's predictions were validated with respect to the measured contact forces for various gait patterns. Particularly, bouncy (“bouncy4” —at each step landing on the front of the foot rather than the heel, causing a bouncy-like walking), crouch (“crouch_og2” —walking while crouching down), forefoot strike (“mtpgait2” —walking on toes), normal (“ngait_og1”), and smooth (“smooth1”) gait trials were selected and analyzed through OpenSim software in order to extract the corresponding boundary conditions. The FEM simulating the above gait patterns was used as an input to FEBio. Contact pressures and forces at the tibiofemoral joint were computed through FEA. Indicatively, the produced contact forces in the femoral component-tibial insert interface for crouch and forefoot strike gait trials were compared with their corresponding *in vivo* contact forces recorded by the implant (Figure 8).

The experimental and predicted contact forces are similar for the crouch (crouch_og2 trial) gait as indicated in Figure 8 (left). The root mean square (RMS), mean, and standard deviation (STD) values of the predicted and experimental contact force curves for crouch gait were computed as a way to quantify the similarity. The relative percentile variation (VAR) indicated that RMS, mean, and STD values of predicted and experimental articular contact force curves (Figure 8, left) are much alike (RMS Var: 8.2%, Mean Var: 6.9%, STD Var: 17.8%). The relative percentile variation is defined as “[estimated_value–reference_value]/reference_value.” In this case, the “estimated_value” refers to the predicted force values, and the “reference_value” is obtained from the sensor's force measurements (experimental value, provided by 6th GCC).

Nevertheless, an underestimation of predicted articular contact forces is rather apparent for the forefoot strike gait pattern (mtpgait2 trial, Figure 8, right), something which is also indicated by the difference between this trial's predicted and experimental RMS, mean, and standard deviation values (RMS Var: 20.4%, Mean Var: 18.5%, STD Var: 40.3%). A possible explanation is that the muscle optimization step might cause the underestimation during the extraction of joint reaction forces. The objective of muscle activations squared was used to resolve the muscle redundancy problem (Stanev and Moustakas, 2019). It has been shown that this objective function predicts well muscle activity for healthy cyclic movements (Anderson and Pandy, 2001). However, if the movement is not typical (e.g., toe walking), the central nervous system might adopt a different control strategy. Therefore, different muscle forces might result in different contact forces.

**Figure 9:**
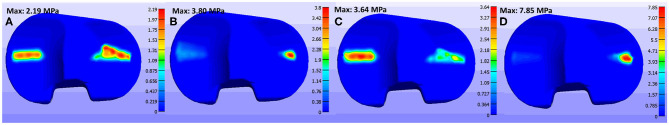
Contact pressure values on tibial insert at **(A)** smooth gait during 1st axial peak force, **(B)** smooth gait during 2nd axial peak force, **(C)** bouncy gait during 1st peak force, and **(D)** bouncy gait during 2nd axial peak force.

Maximum contact pressures for patient's different gait patterns at 1st (loading response—20% stance) and 2nd (pre-swing—80% stance) vertical peak contact forces were also calculated (Table 1). The contact pressure distribution on the tibial insert during smooth and bouncy gait is illustrated in Figure 9. Maximum contact pressure values were observed at bouncy gait during 2nd peak contact force (around 7.85 MPa, Figure 9D) and the lowest stresses during smooth gait pattern (Figures 9A,B). The abrupt changes in joint flexion and axial forces during a bouncy-like walking lead to increased contact forces. Thus, this rise in tibiofemoral contact forces induces higher contact pressures. On the contrary, smooth walking is expected to deplete the pressure distribution at the knee joint. Overall, the observed contact pressures during the patient's different gait patterns are rather satisfactory, since according to related literature, an acceptable range of contact pressure values is ~5–20 MPa (Sharma et al., 2007; Shashishekar and Ramesh, 2007; Moewis et al., 2018).

### 3.2. The Influence of Ligament Parameters on Contact Pressures

Parameters, such as ligament origin points position, reference strain, and ligament stiffness were used to conduct a sensitivity analysis of the model's response. Table 2 summarizes the maximum contact pressures during normal gait at first (20% stance) and second (80% stance) vertical peak forces after changing the ligament properties. The relative percentile variation of contact pressures from the reference configuration is in Table 2.

**Table 2:** Maximum contact pressures at tibial insert after ligament parameter changes.

**Ligament parameter changes**	**1st Axial peak force (Mpa)**	**Var %**	**2nd Axial peak force (Mpa)**	**Var %**
Reference configuration	2.93	0.00	6.22	0.00
MCL+LCL AP 5 mm	2.84	−3.07	5.22	−16.08
MCL anterior 5 mm	2.74	−6.48	5.14	−17.36
MCL posterior 5 mm	2.93	0.00	6.34	1.93
LCL anterior 5 mm	2.91	−0.68	6.44	3.54
LCL posterior 5 mm	2.93	0.00	6.08	−2.25
MCL+LCL ML 5 mm	2.93	0.00	6.33	1.77
MCL medial 5 mm	2.93	0.00	6.89	10.77
MCL lateral 5 mm	2.93	0.00	6.33	1.77
LCL medial 5 mm	2.93	0.00	6.08	−2.25
LCL lateral 5 mm	2.93	0.00	6.08	−2.25
MCL+LCL PD 5 mm	2.93	0.00	6.68	7.40
MCL proximal 5 mm	2.93	0.00	7.06	13.50
MCL distal 5 mm	2.92	0.00	5.69	−8.52
LCL proximal 5 mm	2.93	0.00	6.08	−2.25
LCL distal 5 mm	2.93	0.00	6.08	−2.25
MCL+LCL *ϵ*_*r*_ −5%	2.93	0.00	6.28	0.96
MCL *ϵ*_*r*_ −5%	2.93	0.00	6.26	0.64
LCL *ϵ*_*r*_ −5%	2.93	0.00	6.08	−2.25
MCL+LCL *ϵ*_*r*_ +5%	2.82	−3.75	5.71	−8.20
MCL *ϵ*_*r*_ +5%	2.93	0.00	6.11	−1.77
LCL *ϵ*_*r*_ +5%	2.93	0.00	6.08	−2.25
MCL+LCL *ϵ*_*r*_ −10%	2.93	0.00	6.28	0.96
MCL *ϵ*_*r*_ −10%	2.93	0.00	6.35	2.09
LCL *ϵ*_*r*_ −10%	2.93	0.00	6.08	−2.25
MCL+LCL *ϵ*_*r*_ +10%	2.93	0.00	6.27	0.80
MCL *ϵ*_*r*_ +10%	2.93	0.00	6.71	7.88
LCL *ϵ*_*r*_ +10%	2.93	0.00	6.08	−2.25
MCL+LCL k −10%	2.93	0.00	6.28	0.96
MCL k −10%	2.93	0.00	6.21	−0.16
LCL k −10%	2.93	0.00	6.08	−2.25
MCL+LCL k +10%	2.93	0.00	6.21	−0.16
MCL k +10%	2.93	0.00	6.21	−0.16
LCL k +10%	2.93	0.00	6.08	−2.25

*Ligament origin positions were translated by 5 mm in the anterior-posterior (AP), medial-lateral (ML), or proximal-distal (PD) direction with respect to femur and tibia. Reference strain (*ϵ*_r_) was modified by ±5 and ±10% from reference value. Stiffness (k) was changed ±10% from its initial value*.

Both ligament attachment points of MCL and LCL were translated by the same amount of 5 mm, and in the same anterior-posterior (AP), medial-lateral (ML) or proximal-distal (PD) direction with respect to femur and tibia, so that ligaments' rest-length, *L*_0_, would remain unchangeable, according to Innocenti et al. (2011). Moreover, ligament reference strain *ϵ*_*r*_ was modified based on the configuration of Esrafilian et al. (2020), where MCL's and LCL's pre-tension deviates ±5 and ±10% from the reference value. Finally, ligament stiffness *k* was changed ±10% from its initial value. The above alterations were applied on one of the two ligaments of the model (MCL or LCL) or on both as indicated in Table 2.

From this sensitivity analysis, we can infer that contact pressures, at both axial peak contact forces, were mostly affected by the anterior translation of the MCL ligament. In particular, anterior MCL translation by 5 mm caused a 6.48% decrease of contact pressures at 1st maximum peak force and a 17.36% decrease at 2nd axial force, while translating LCL in the anterior direction by the same amount caused a slight increase in contact pressures. A similar trend of influence of anterior-posterior (AP) MCL and LCL translations on load distributions of different implant types were also observed in Innocenti et al. (2011), where a 5 mm MCL repositioning in the anterior direction caused substantial alterations in maximum tibiofemoral contact forces. In contrast, the anterior translation of LCL increased peak forces at the tibial site in different configurations. The same research team in one of their next works showed that for different implant designs, ligament shifts, as well as implant malpositioning, also affect TKA kinematics (Pianigiani et al., 2012).

Nevertheless, a rise in pressures at 2nd axial peak contact forces was occasioned by changing the position of ligaments' attachment points in the medial-lateral (ML) and proximal-distal (PD) direction by 5 mm as well. Notably, medial and proximal MCL translation increased contact pressures by 10.77 and 13.50% during 2nd maximum contact forces, respectively.

Modifications in ligaments' reference strain led to negligible variations in pressure concentration during 1st axial peak forces. A reduction of 8.20% at 2nd peak force was recorded when both ligaments' reference strain increased by 5%. Altering MCL's reference strain by +10% causes a 7.88% increase in contact pressures at 2nd peak force. Similarly, the authors of Esrafilian et al. (2020) observed rises at maximum principal stresses at the 2nd axial peak force by increasing the reference strain in MCL by 10%, while likewise changing the LCL reference strain did not induce significant modifications in maximum pressure.

### 3.3. The Influence of Implant Alignment on Contact Pressures

The femoral component's alignment was modified following common practices adopted by other authors (Liau et al., 2002; Pang et al., 2016). In particular:

- Femoral component was internally/externally rotated in the range of [0, 5°] relative to tibial insert.
- Femoral component was given varus/valgus tilt in the range of [0, 5°] relative to tibial insert.

The maximum contact pressures after implant misalignment at 1st and 2nd axial peak forces, during the patient's normal gait pattern, are illustrated in Table 3. The relative percentile variation of contact pressures during malaligned conditions from the reference value is reported in Table 3.

**Table 3:** Maximum contact pressures at different femoral component misalignment conditions.

**Alignment changes**	**1st Axial peak force (Mpa)**	**Var %**	**2nd Axial peak force (Mpa)**	**Var %**
Reference configuration	2.93	0.00	6.22	0.00
Varus tilt 1°	2.88	−1.71	7.83	25.88
Varus tilt 3°	2.69	−8.19	7.00	12.54
Varus tilt 5°	3.14	7.16	10.10	62.38
Valgus tilt 1°	2.78	−5.12	6.01	−3.38
Valgus tilt 3°	2.73	−6.83	5.49	−11.74
Valgus tilt 5°	2.68	−8.53	4.72	−24.12
Internal rotation 1°	3.87	32.08	7.14	14.79
Internal rotation 5°	8.98	206.48	14.50	133.12
External rotation 1°	2.96	1.02	6.25	0.48
External rotation 5°	4.92	67.82	8.39	34.89

*The femoral component was internal/external, and varus/valgus rotated in the range of [0, 5°] relative to tibial insert*.

The femoral component's valgus tilt in various misalignment angles caused a minimal decrease in 1*st* peak contact forces. Also, aligning the femoral implant with a varus tilt relative to the tibial insert resulted in significant increases in contact pressures during 2nd axial peak force (up to 62.38% at 5°). Also reported in Liau et al. (2002), a rise in maximum contact pressures under varus tilt (1, 3, and 5°) of the femoral component relative to the tibial component was observed. Similarly, the authors of Innocenti et al. (2016) observed that configurations with more severe femoral component varus alignment (e.g., 4 and 6°) lead to an increase of contact stresses on tibial insert. Nonetheless, a reduction in contact pressures (down to 24.1%) was noted in our results by rising valgus tilting angles.

Maximum contact pressures were observed while rotating the femoral compartment internally by 5°, where an increase of 206.48 and 133.12% was noted in both peak contact forces, respectively. Contrariwise, Liau et al. (2002) and Xie et al. (2017) showed that the femoral component's internal rotation in TKR alignment produces slightly lower maximum contact pressures than reference configuration. Furthermore, a rise of 67.92% on 1st and 34.89% on 2nd maximum axial forces was achieved by rotating femoral component 5° externally. These results also contrast with Xie et al. (2017), where it is supported that femoral external rotation alignment can lead to reduced peak tibiofemoral contact pressure concentration.

Nevertheless, we can assume a similarity between our observations during the femoral component's internal and external rotation and the ones of Yazdi et al. (2016). In this study, the effect of tibial rotation on contact pressure distribution on knee medial and lateral compartments was studied. The results of Yazdi et al. (2016) indicated that greater tibial internal and external angles cause an increase in medial and lateral compartment contact pressures. Notably, an internal tibial rotation of 15 and 30° and an external tibial rotation of 30° increased significantly contact pressures over the medial knee compartment, while 15 and 30° external tibial rotation increased stresses at the lateral knee compartment.

The observed increase in contact pressures while rotating the femoral component internally, externally, and given varus tilt may be due to the patient's initial implant positioning. In particular, the patient's femoral component was initially given a slight varus tilt, which is unavoidably leading to higher contact pressures at the right lateral side of the tibial plateau (Figure 9). By comparing the patient's pre-operational and post-operational image data, it was assumed that the alignment of the arthritic (right) knee was changed from a valgus to a neutral/mechanical one. This means that the TKR implant was positioned in such a way to create a straight limb with a perpendicular tibiofemoral joint line (Schiraldi et al., 2016). Thus, since the initial knee joint anatomy was not considered, higher load distributions were produced at the tibial plateau. Different studies (Bellemans et al., 2012; Lording et al., 2016; Schiraldi et al., 2016; Riviére et al., 2018) state that even though the mechanical alignment is the most common approach in TKA, which is preferred for prolonging implant survivorship, other more personalized solutions may improve the aftereffects of the surgery, especially in patients with unique/non-neutral knee alignment.

After that, the presence of high contact pressures at the tibial insert intensifies by introducing a small misalignment (internal, external, and varus tilting), combined with implanted knee's initial varus tilt. Possible mistakes during implant-bone alignment may also deteriorate this phenomenon. Thus, the latter remarks accumulate error in the computation of contact pressures at the tibiofemoral joint and explain the significant increase in contact pressures, especially during the femoral component's 5° degrees of internal rotation.

## 4. Discussion

Data from the 6th Grand Challenge data set were utilized throughout the proposed framework. Motion capture data were used as boundary conditions for the model. Simple python scripts were created to preprocess the gait GCC data, including format conversions, data filtering, and rotation of GRF vectors. Moreover, the extraction of boundary conditions was done through OpenSim analyses. These data were sufficient in order to reproduce contact forces comparable with the experimental ones.

This work focused on the creation of individualized models. Prediction of contact pressures and other estimates are influenced by patient-specific geometries, joint definitions, and loading conditions (Xu et al., 2020). Personalized bone geometries were extracted by pre-operational subject-specific CT data. Moreover, patient-specific alignment between implant-bone geometries was achieved through post-operational CT images. Not considering subject-specific parameters (e.g., geometry and material properties) may lead to errors between model predictions and experimental data as stated in Kiapour et al. (2014). Therefore, by moving in the direction of patient-specific modeling, we can better account for these specificities in our model.

The implant 3D geometries used in the proposed pipeline were also included in the GCC data set. A good practice would have been to create new 3D geometries of the TKR implant parts through segmentation of the post-operational CT images and not use the GCC ones. Although, the reflection of the metallic implant parts was blurring the post-operational images to the extent that it was impossible to segment the implant geometries. The blur in the images may have affected the alignment procedure's precision, resulting in slightly higher load predictions at the joint. Finally, geometry processing software was used for correcting artifacts in implant-bone geometries and create their isotropic and volumetric meshes. The geometry preparation step of the proposed pipeline is rather significant to create meshes suitable for FEA.

In this study, we developed a framework for constructing FE models, which can be used to investigate the biomechanical behavior of various patient-specific implanted knee joints through FEA. The proposed framework's main benefit is that FEM can be rapidly prototyped in a semi-automatic manner using custom-built Python scripts. There is a specific pattern of modeling steps for the particular problem, which can be automated and generalized. Compared to manual approaches (Kiapour et al., 2014; Marra et al., 2015; Navacchia et al., 2016; Shu et al., 2018), the benefits are reduction in modeling effort and time, consistency between different patient-specific models, and reproducibility of results (Erdemir et al., 2019). Therefore, manual modeling may take more than 3 h from a user perspective, whereas the current approach defines the FEM in <3 min.

The FEM's predictions were validated based on *in vivo* measured contact forces for various gait trials provided in the 6th Grand Challenge data set. The produced knee contact forces via FEA were mostly in accordance with the corresponding *in vivo* recordings for different individualized gait trials, which is a good indication for validity. Moreover, observed contact pressures at the tibial insert surface are rather satisfactory according to related literature (Sharma et al., 2007; Shashishekar and Ramesh, 2007; Moewis et al., 2018).

The use of scripting facilitates the automation of sensitivity analysis. The sensitivity analysis was conducted to assess the influence of ligament properties and implant positioning on the tibial insert's contact pressures and determine possible uncertainties on the models produced by the framework. Models with modifications in MCL and LCL's characteristics, namely, origin ligament positions, reference strain *ϵ*_*r*_, and ligament stiffness *k* were used for this analysis. Furthermore, FEMs with differences in femoral component placement (internal/external and varus/valgus rotation) were created and tested. Except the influence of femoral component's positioning in the sensitivity of contact pressures and subsequently in implant survival, Innocenti et al. (2016) underline that changes in tibial component's positioning greatly affects the contact loads at the tibiofemoral area. Moreover, in Ritter et al. (2011), the important role of the tibial component's malalignment in frequent revision TKR rates is also discussed. Therefore, the effects of tibial component's alignment on tibiofemoral contact mechanics, which were not explored in the present study, is proposed as future work.

The findings of the above sensitivity analysis indicated that fluctuations in ligaments' origin point positioning affected more the pressures at the tibial insert area; with anterior MCL translation causing the most decrease (−17.36% at 2nd axial force) and proximal MCL translation causing the most increase in contact pressures (10.77% during 2nd axial peak force). However, parameters, such as ligaments' reference strain and stiffness did slight alterations to the tibiofemoral joint's contact pressures. Maximum contact pressures were noted during the femoral component's internal rotation, where an increase of 206.48% at 1st axial peak force and an increase of 133.12% at 2nd axial force were observed when the femoral part was rotated by 5°.

The proposed personalized model poses a few limitations to the study of knee kinematic and dynamic behavior. First, the tibial tray was considered as a rigid body. This simplification reduces the simulation's precision and contrasts with a real-life knee prosthesis, where the tibial tray is usually a metallic, rigid, but still deformable object. However, the tibial tray has a much higher modulus of elasticity than the tibial insert. So, its deformation is negligible during knee function compared to the tibial plateau. Therefore, since we are interested in investigating the tibial insert's contact pressures and forces, modeling the tibial tray otherwise would only increase the procedure's execution time.

In continuity, the FEM has only three out of total of six DOFs of the knee joint enabled, namely, joint–distraction (z-axis displacement), abduction–adduction (y-axis rotation), and the flexion–extension (x-axis rotation) motion. Joint–distraction and abduction–adduction aid in better contact detection at the joint interface, leading to more accurate contact pressure computations during flexion–extension. Enabling these DOFs can help simulate knee mechanics while keeping the model as simple as possible. Although enabling z-rotation, x- and y-axis displacements would make the model function more complex and even lead to convergence problems.

Finally, the patella along with the patellar implant was excluded from this research. The examination of patellofemoral mechanics and contact pressures and stresses requires modeling muscles, which is out of the scope of the present work. The extension of this FEM with muscles that would support a patellar bone and implant is proposed as future work, along with the validation of the model based on data from more patients with osteoarthritis.

This project lays the foundation for extended sensitivity studies on TKR with more than one subject. The automatic construction of TKR models facilitates both the production and testing of a vast amount of subject-specific models and poses as a new approach to develop custom-made implants. With the evolution of 3D printing technologies, the predefined design of implants will transition to a more patient-specific approach. Thus, the production of custom-made implants designed via simulation could be facilitated, and the need for more accurate model representations will rise. Moreover, the proposed framework may lead research closer to optimizing a personalized surgery by evaluating the best surgical parameters before TKA. For instance, a trial of how to conduct bone resections and predict the effects of implant alignment on a specific patient's implanted knee can be made pre-operatively through simulation. Thereby, the causes of TKR failures, such as misalignment, infection, which would result in pain, material loss, and overall deterioration of the tibial plateau and implant fractures, can be further researched.

## 5. Conclusion

The proposed methodology constitutes a complete framework containing guidelines on the development and individualization of a FEM, extraction of knee bone geometries through segmentation of patient-specific CT data, alignment of implant-bone components, and geometry processing techniques. This study's main contribution is the automation of the FEM development process, which speeds up the proposed framework and makes it viable for practical applications. A stable modeling framework (script) aids in making the modeling choices consistent and easy to reproduce by other users (Erdemir et al., 2019). Furthermore, the validation process indicated that FEMs produced by this pipeline could predict the contact forces measured by e-Tibia's sensor (i.e., force data from the 6th GCC dataset). Sensitivity analysis was used to study the effect of ligament and position parameter alterations in tibiofemoral contact pressures. In conclusion, the proposed framework's models can be further used in the pre-clinical optimization of customized total knee replacement operations.

## Data Availability Statement

Publicly available datasets were analyzed in this study. This data can be found at: https://simtk.org/frs/?group_id=413. Data for Sixth Competition-Institutional review board approval and subject informed consent were obtained for all de-identified competition data being released by the research team who made available this data set. The competition data and models can be used for research, publication, and grant proposals' purposes.

## Author Contributions

IL and DS contributed to the conception, software implementation, and design of the study. IL wrote the first draft of the manuscript. DS and KM supervised the manuscript. All authors contributed to manuscript revision and approved the submitted version.

## Conflict of Interest

The authors declare that the research was conducted in the absence of any commercial or financial relationships that could be construed as a potential conflict of interest.
